# Low fat cake formulation and characterization using wheat germ, rice bran, and pumpkin seeds as a fat replacers

**DOI:** 10.1038/s41538-026-01011-2

**Published:** 2026-07-24

**Authors:** Maha I. K. Ali, Seham Y. Gebreil, Saadia M. Hashem

**Affiliations:** 1 Department of Special Food and Nutrition, Food Technology Research Institute, Agricultural Research Center, Giza, Egypt; 2 Crops Technology Research Department, Food Technology Research Institute, Agricultural Research Center, Giza, Egypt; 3 Food Science and Technology Department, Faculty of Agriculture (EL-Shatby), Alexandria University, Alexandria, 21545 Egypt

**Keywords:** Biochemistry, Biotechnology, Health care, Plant sciences

## Abstract

In the context of increasing interest in healthy and sustainable nutrition, the food industry is challenged to develop innovative products that combine high nutritional quality with consumer acceptance. Therefore, the present study aimed to investigate wheat germ (WG), pumpkin seeds (PS), and rice bran (RB) powders as natural fat replacers in cakes, incorporated at levels of 60% WG, 60% PS, and 40% RB. The formulated cakes were evaluated for physicochemical properties, mineral and vitamin composition, color attributes, texture profile, microbial stability, and sensory quality. Cakes containing WG showed the highest levels of protein, dietary fiber, phenolic and flavonoid contents, as well as elevated vitamins (E and B-complex) and minerals. These samples also exhibited increased firmness, gumminess, and chewiness compared to the control. Cakes with RB showed higher antioxidant activity and improved mineral content. No significant differences were observed in overall sensory acceptability among all formulations. Moreover, all fortified cakes demonstrated improved microbial stability during storage. Overall, wheat germ, pumpkin seeds, and rice bran powders can be effectively utilized as natural fat replacers to produce nutritionally enhanced, antioxidant-rich, and microbiologically stable cake products with acceptable sensory properties.

## Introduction

Nowadays, consumers have grown more conscious about their dietary selections, seeking foods that offer health benefits and deliver appealing taste, texture, and appearance. Baked goods, particularly bread, biscuits, and cakes, are among the most widely consumed food items worldwide due to their accessibility, convenience, and long shelf life^1^. However, these products are typically prepared using refined wheat flour, which is deficient in protein and essential amino acids. Furthermore, its high gluten content can cause adverse reactions in gluten-sensitive individuals. Alongside these nutritional concerns, there is a growing awareness of the negative health impacts associated with excessive consumption of fats and sugars, further motivating the development of healthier bakery products^2^.

Simultaneously, the agriculture and food processing industries generate substantial quantities of by-products and waste each year, creating environmental challenges related to disposal and landfill management^3^. Food industry waste is generated at multiple points in the supply chain, from harvesting and sorting to processing^4–6^. Increasingly, attention is being given to the valorization of these by-products as cost-effective sources of bioactive compounds, including phenolics, antioxidants, vitamins, and minerals, protein, and dietary fiber^7^. Incorporating these nutrient-rich materials into human diets improves nutritional quality and mitigates environmental impacts^8^. Despite this potential, agricultural by-products remain underutilized, with approximately 14% of global food loss occurring during post-harvest stages^9^.

Among such by-products, rice bran (RB), a residue from rice milling that is the world’s third most consumed cereal, stands out for its nutritional richness. Constituting approximately 12% of the total grain weight, RB comprises the outer layers of the seed, including the pericarp, tegmen, and aleurone layer, resulting in nearly 68 million tons of residual material annually^9,10^. Compared to the starchy endosperm, these layers contain higher concentrations of dietary fiber, proteins, lipids, minerals, vitamins, and bioactive compounds like ferulic acid, tocopherols, and γ-oryzanol, making RB a valuable ingredient for functional foods^11^.

Wheat germ (WG), which constitutes only about 2–3% of the wheat grain, remains relatively underexplored despite its nutritional significance. Although it represents a small fraction of the grain, WG is a concentrated source of functional compounds, including bioactive peptides, polyunsaturated fatty acids, tocopherols, and dietary fiber, all of which possess strong antioxidant and anti-inflammatory properties. The anti-inflammatory, anticancer, and lipid-lowering effects of WG have been demonstrated in both animal models and humans. Furthermore, bioactive peptides derived from WG have demonstrated the ability to inhibit ROS production, alleviate oxidative stress, and enhance mitochondrial energy metabolism. Its richness in polyunsaturated fatty acids also contributes to enhanced bone metabolism and increased bone mass. Moreover, the dietary fiber in WG exhibits prebiotic potential with notable antidiabetic activity. In addition to these benefits, WG contains important amino acids, fatty acids, vitamins, minerals, tocopherols, and phytosterols, which makes it a great addition to cereal products that usually lack micronutrients and dietary fiber^12,13^_._

Similarly, pumpkin (*Cucurbita spp*.) and its seeds are gaining attention for their exceptional nutritional value and phytochemical diversity^14^. The nutritional benefits and health-promoting properties of pumpkin seeds have gained considerable attention recently. Pumpkin seeds are a rich source of essential minerals, including magnesium, zinc, copper, molybdenum, selenium, and several other trace elements^15^. Various phytochemicals, including polysaccharides, phenolic glycosides, non-esterified fatty acids (NEFA), and proteins, have been extracted from pumpkin leaves and germinating seeds^16^. Although often regarded as waste, pumpkin seeds are in fact rich in bioactive compounds with significant nutraceutical potential^17^. Naturally occurring bioactive compounds such as carotenoids, tocopherols, and sterols exhibit various biological activities in vivo, aiding in the prevention of cancer, diabetes, and hypertension^18^. Many studies have shown that pumpkin seeds are very high in antioxidants^19^, which also have phenolic compounds like saponins, flavonoids, and alkaloids^20^. Notably, pumpkin seeds possess the highest antioxidant levels among the different parts of the pumpkin^21^.

Cakes are among the most popular bakery products, yet their high levels of saturated and trans fats raise health concerns related to obesity and cardiovascular diseases. Growing consumer awareness and nutritional regulations have intensified the demand for reduced-fat bakery items^22^. However, replacing fat without compromising texture, flavor, and mouthfeel remains a major challenge. Fat substitutes, especially those based on protein and carbohydrates, can replicate some of the functional and sensory roles of fat while also reducing calorie content^23^. Recently, attention has focused on natural, plant-derived ingredients as promising fat replacers. By-products such as wheat germ (WG), rice bran (RB), and pumpkin seed (PS) powders are rich in fiber, antioxidants, and bioactive compounds, offering both nutritional and functional benefits.

Despite the well-documented nutritional and functional properties of wheat germ, rice bran, and pumpkin seeds, most previous studies have investigated these by-products individually as enrichment ingredients rather than as direct fat replacers in bakery products. Moreover, limited research has focused on their application at relatively high fat replacement levels, where maintaining acceptable texture, sensory quality, and storage stability remains challenging. In addition, comparative studies evaluating the performance of these cereal by-products under identical formulation and processing conditions within the same cake matrix are scarce. Therefore, a clear research gap exists regarding the feasibility and effectiveness of directly comparing wheat germ, rice bran, and pumpkin seeds powders as high-level natural fat replacers in cake formulations. Accordingly, the present study aimed to comparatively evaluate wheat germ (60%), pumpkin seeds powder (60%), and rice bran (40%) as natural fat replacers in cake formulations, with emphasis on their effects on physicochemical properties, nutritional composition, antioxidant activity, textural and sensory attributes, and storage stability. Unlike conventional single-ingredient substitution studies, the present work adopts a multi-ingredient comparative framework under identical formulation and processing conditions, enabling direct evaluation of how compositional differences among wheat germ, rice bran, and pumpkin seeds powder influence their functional performance as high-level fat replacers. This integrated approach provides clearer mechanistic insight and practical guidance for selecting suitable natural fat alternatives in bakery applications.

## Results and discussion

### Physicochemical properties of wheat germ, pumpkin seeds and rice bran

Table 1 presents the physicochemical properties of wheat germ, pumpkin seeds, and rice bran. The results indicate that pumpkin seeds surpass both wheat germ and rice bran in terms of crude protein, crude fat, and energy content, with values of 32.00%, 25.29%, and 459.21 kcal/100 g, respectively. Rice bran, on the other hand, has more moisture, crude fiber, and ash than the other two materials (8.98%, 11.03%, and 13.49%, respectively). Additionally, rice bran is characterized by the highest total carbohydrate content (59.23%). When compared with previous studies, in fact, the results of our study were lower than those of Polyzos et al.^24^, who documented that pumpkin seeds contained 37.67% protein, 42.74% crude fat, 3.52% ash, 16.07% total carbohydrates, and an energy value of 599.62 kcal/100 g. Additionally, Rodríguez-Fernández et al.^25^ found that wheat germ comprised 25.75% protein, 7.64% total fat, 4.30% ash, 24.90% fiber, 29.05% total carbohydrates, and 8.37% moisture. As for rice bran, Ayoub et al.^26^ reported a composition of 12.66% protein, 12.33% total fat, 9.86% ash, 13% fiber, and 9.20% moisture. Although the proximate composition values obtained in the present study differ from those reported in previous literature, such variations are commonly attributed to several factors. Differences in plant variety or cultivar, geographical origin, environmental conditions during growth, agricultural practices, degree of seed maturity, and post-harvest handling all significantly influence the chemical composition of cereal by-products. In addition, variations in processing methods such as drying, grinding, and storage conditions can also affect moisture, fat, protein, and ash contents. Therefore, the discrepancies observed between the present findings and earlier studies fall within the expected range of natural and processing-related variations reported for these raw materials.

**Table 1:** Physicochemical properties of wheat germ, pumpkin seeds and rice bran

Physicochemical properties	Raw material		
	WG	PS	RB
Chemical composition			
Moisture (%)	5.99 ± 0.02^b^	6.02 ± 0.01^b^	8.98 ± 0.03^a^
Crude protein (%)	28.08 ± 0.01^b^	32.00 ± 0.02^a^	7.71 ± 0.02^c^
Crude fat (%)	9.63 ± 0.68^b^	25.29 ± 0.54^a^	8.54 ± 0.46^b^
Crude fiber (%)	2.53 ± 0.04^c^	10.02 ± 0.02^b^	11.03 ± 0.02^a^
Total ash (%)	4.68 ± 0.01^c^	6.79 ± 0.01^b^	13.49 ± 0.02^a^
Total carbohydrates (%)	55.08 ± 0.65^b^	25.90 ± 0.44^c^	59.23 ± 0.48^a^
Energy value (kcal/100 g)	419.31 ± 3.59^b^	459.21 ± 2.15^a^	344.62 ± 2.27^c^
Bioactive compounds			
Total phenolic content (mg GAE/100 g)	87.14 ± 0.51^b^	8.66 ± 0.32^b^	4.49 ± 0.39^c^
Total flavonoid content (mg RE/100 g)	20.76 ± 0.54^a^	3.14 ± 0.30^c^	5.37 ± 0.37^b^
DPPH scavenging activity (%)	68.31 ± 0.61^b^	41.46 ± 0.94^c^	74.68 ± 0.85^a^
Physical properties			
Water holding capacity (%)	264.00 ± 1.00^a^	255.75 ± 0.58^c^	229.00 ± 3.00^b^
Oil holding capacity (%)	236.67 ± 1.52^a^	193.89 ± 2.65^c^	227.00 ± 3.00^b^

*WG* wheat germ, *PS* pumpkin seeds, *RB* rice bran.

Values represent the means ± SD (on dry weight basis) of three independent replicates. Means within the same row followed by different letters are significantly different (*p* < 0.05).

Table 1 illustrates the bioactive compound content and DPPH radical scavenging activity of wheat germ, pumpkin seeds, and rice bran. Wheat germ showed significantly higher levels of total phenolics (87.14 mg GAE/100 g) and total flavonoids (20.76 RE/100 g) compared to pumpkin seeds and rice bran, which recorded lower values of 8.66 and 4.49 mg GAE/100 g and 3.14 and 5.37 mg RE/100 respectively. Conversely, rice bran exhibited the highest DPPH radical scavenging activity (74.68%), despite its lower total phenolic content, suggesting that the antioxidant efficiency of rice bran may be attributed to the presence of specific phenolic profiles and synergistic effects of bioactive compounds rather than total phenolic concentration alone. Pumpkin seeds demonstrated the lowest antioxidant activity (41.46%). Previous studies have also demonstrated that wheat germ extracts are rich in phenolic compounds. However, the reported values vary considerably depending on the extraction solvent, extraction conditions, and the units and basis used to express the results^27^. On the contrary, Hussain et al.^21^ stated that in the various parts of pumpkin, the seeds exhibited the highest total phenolic content and the peel the lowest. This evidence establishes the fact that pumpkin seeds can be regarded as an important source of phenolic compounds having fairly good antioxidant potential. Measurement of total phenolics was based on gallic acid equivalents due to its acid stability and its strong reactivity with the Folin-Ciocalteu reagent. According to what is stated by Abd Razak et al.^28^ the higher content of phenolics found in pumpkin fruits and seeds can be attributed to the role of these structures as primary sites of plant metabolism and secondary metabolite production. Furthermore, rice bran is known to have strong antioxidant activity, which is largely attributed to its phytochemicals and bioactive peptides. Wanyo et al.^29^ discovered that rice bran possesses better antioxidant capacity, which reaches 87.93%, thus proving its value as a functional food component.

The physical properties of wheat germ, pumpkin seeds, and rice bran are presented in Table 1. Wheat germ exhibited significantly higher water-holding capacity 264.00% and oil-holding capacity 236.67% compared to pumpkin seeds and rice bran. The functional properties of the materials created direct changes to both the batter structure and the final cake quality. The high water-holding capacity likely enhanced moisture retention within the cake matrix, which resulted in better cake moisture retention. The elevated oil holding capacity may have partially compensated for the reduced lipid content, which led to improved mouthfeel and crumb softness in WG-containing cakes. The increased oil-holding capacity of wheat germ has been attributed to the exposure of hydrophobic groups during protein unfolding, which enhances lipid interaction. The protein–lipid interactions in this system create stabilization for air cells, which reside within the batter system, and they ultimately determine the cake volume and texture^30^.

Similarly, da Rocha Lemos Mendes et al.^31^ stated about the water-binding and oil-absorption capacities of rice bran suggest its ability to retain moisture and interact with lipids. However, the comparatively lower values relative to wheat germ may explain the denser structure and firmer texture observed in RB cakes at higher substitution levels^30^.

### Chemical composition and bioactive compounds of cake samples

**Fig. 1:**
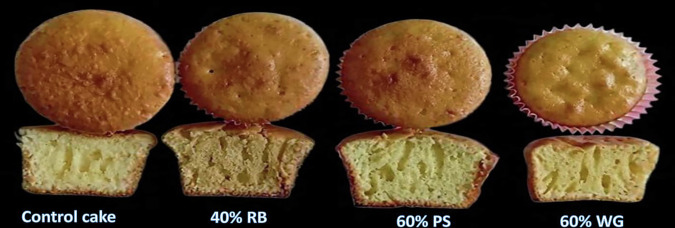
Cake samples. Control cake; 60% WG cake containing 60% wheat germ, 60% PS cake containing 60% pumpkin seeds, 40% RB cake containing 40% rice bran.

The cake samples produced in this study are illustrated in Fig. 1. The incorporation of wheat germ (WG), pumpkin seed powder (PS), and rice bran (RB) as natural fat replacers enabled the development of functional cake formulations enriched with valuable nutrients derived from cereal by-products that are often discarded. Beyond improving nutritional quality, this approach supports sustainability by valorizing food processing residues. As shown in Fig. 1, the addition of these ingredients influenced cake appearance, resulting in a darker crust and slightly denser crumb compared to the control, which can be attributed to their higher fiber and mineral contents, as reported for the raw materials in Table 1.

Chemical composition and bioactive compounds of cake samples are presented in Table 2. Moisture content ranged from 19.33% to 22.00%, with the highest value (*P* < 0.05) recorded in the cake formulated with 60% wheat germ, while the control exhibited comparatively lower moisture content. This variation can be attributed to differences in water-binding capacity among the incorporated by-products. Wheat germ contains appreciable levels of protein and dietary fiber (Table 1), which enhance water absorption and retention within the cake matrix through hydrogen bonding and structural entrapment mechanisms. In addition, the presence of fiber may modify water distribution between free and bound fractions, thereby influencing the measured moisture content. These differences are therefore associated with compositional characteristics of the fat replacers rather than processing variability. Similar trends have been reported by da Rocha Lemos Mendes et al.^31^ found moisture contents ranging from 24.5% in the control to 27.4–33.7% in cakes fortified with defatted rice bran (DRB). Due to its high fiber and protein content.

**Table 2:** Chemical composition and bioactive compounds of cake samples

Component	Cake samples			
	Control sample	60% WG	60% PS	40% RB
Chemical composition				
Moisture (%)	19.33 ± 0.02 ^d^	22.00 ± 0.02^a^	21.75 ± 0.01^b^	20.69 ± 0.02^c^
Crude protein (%)	6.70 ± 0.01 ^d^	11.36 ± 0.01^b^	12.05 ± 0.01^a^	7.24 ± 0.01^c^
Crude fat (%)	19.95 ± 0.64^a^	12.59 ± 0.98^bc^	14.08 ± 0.28^b^	10.97 ± 0.77^c^
Crude Fiber (%)	0.23 ± 0.01 ^d^	0.60 ± 0.01^c^	1.42 ± 0.01^a^	1.18 ± 0.01^b^
Total ash (%)	1.26 ± 0.01 ^d^	1.98 ± 0.01^c^	2.51 ± 0.01^b^	2.74 ± 0.01^a^
Total carbohydrates (%)	71.86 ± 0.65^c^	73.47 ± 0.97^b^	69.94 ± 0.29 ^d^	77.87 ± 0.78^a^
Energy values (kcal/100 g)	493.79 ± 3.24^a^	452.63 ± 4.95^b^	454.68 ± 1.35^b^	439.17 ± 3.85^c^
Bioactive compounds				
Total phenolic content (mg GAE/100 g)	24.56 ± 0.11 ^d^	70.28 ± 0.06^a^	28.27 ± 0.04^b^	27.24 ± 0.12^c^
Total flavonoid content (mg RE/100 g)	0.86 ± 0.01 ^d^	11.99 ± 0.10^a^	2.07 ± 0.10^c^	2.66 ± 0.04^b^
DPPH scavenging activity (%)	8.36 ± 0.08 ^d^	36.77 ± 0.10^b^	31.71 ± 0.17^c^	58.65 ± 0.14^a^

*60% WG* cake containing 60% wheat germ, *60% PS* cake containing 60% pumpkin seeds, *40% RB* cake containing 40% rice bran. Data are presented as mean ± SD (*n* = 3). Different letters indicate significant differences at *p* < 0.05.

Table 2 shows that the protein content of the cakes ranged from 6.70% to 12.05%, with the highest protein content in the cake made with 60% pumpkin seeds as a fat replacer, while the control cake was found to have lower protein content. This significant increase is directly related to the intrinsic chemical composition of pumpkin seeds, which exhibited the highest crude protein content (32.00%) among the studied raw materials (Table 1). The substitution of corn oil with pumpkin seeds powder therefore introduced a substantial amount of plant protein into the cake formulation, enhancing its nutritional value compared to the control and other treatments. In contrast, the relatively lower protein content observed in cakes containing rice bran is consistent with the lower protein level of rice bran compared to pumpkin seeds. These findings are consistent with those of Singh and Kumar^30^, indicated that pumpkin seeds are a major source of fat and protein, which can be used as a great resource for combating nutritive deficiencies (like malnutrition) and for the prevention of a variety of health disorders. Pumpkin seeds are also a source of high-quality lipids containing essential and nonessential fatty acids, which consist of ω-3, ω-6, and ω-9 fatty acids. Less protein was found in cake with 40% rice bran (RB). However, Alamsyah et al.^32^ found that the addition of rice bran into the cupcakes increased protein content from 7.15% in the control sample to 8.71%, due to the high protein level in rice bran flour (12.14%), which is considerably higher than that of wheat.

The fat content of the cake samples ranged from 10.97% to 19.95%. The sample with 40% rice bran as a fat replacer had a significantly lower fat content compared to that of the control cake, as well as the other samples containing either 60% pumpkin seeds or 60% wheat germ. This reduction can be attributed to the compositional profile of rice bran (Table 1), which is characterized by high carbohydrate and fiber contents and comparatively lower lipid levels. Replacing a portion of corn oil with rice bran powder effectively diluted the overall fat content of the formulation. Our results align with the findings of da Rocha Lemos Mendes et al.^31^_,_ who reported that the cake containing 40% rice bran had the lowest fat content (14.1%) compared to the control cake (21.5%). Notably, only the 40% DRB formulation exhibited a marked reduction in lipid content, with a decrease of 47% compared to the other formulations.

The results of Table 2 show that the crude fiber level in the cake made with 60% fat replacement with pumpkin seeds (1.41%) was significantly higher than in the control cake (0.23%) and other cakes prepared with 40% rice bran and 60% wheat germ (1.18% and 0.60%, respectively). The highest fiber content was observed in the cake containing 60% pumpkin seed powder, which reflects the naturally high fiber content of pumpkin seeds (10.02%, Table 1). Similarly, the increased fiber level in rice bran containing cakes can be explained by the high dietary fiber content of rice bran (11.03%), contributing to improved nutritional quality and potential functional benefits such as enhanced texture stability and glycemic control. These results correlate with findings by Syed et al.^33^, stating that pumpkin seeds are a fair source of fibers, which vary from 3 to 6%. Fiber has gained profound attention among the industries and researchers due to its functional attributes, as well as its impact on the sensory and rheological properties of the finished product^34^.

As presented in Table 2, the cake containing 40% rice bran as a fat replacer showed the highest (*P* < 0.05) ash and carbohydrate contents, reaching 2.74% and 77.87%, respectively. This finding can be attributed to the inherently high mineral and carbohydrate contents of rice bran. In contrast, the cake formulated with 60% pumpkin seed powder recorded the lowest carbohydrate content, which reflects the higher protein and lipid proportions of pumpkin seeds. Meanwhile, the cake prepared with 60% wheat germ as a fat replacer exhibited a reduced ash content compared to the control and other formulations, consistent with the comparatively lower mineral content of wheat germ. Such variability in composition has been reported to depend on the botanical variety, which explains their successful application in different bakery products, including cakes, cookies, and bread^30^. As per da Rocha Lemos Mendes et al. ^31^, the content of crude fiber was between 2.8% and 6.5%, with the 40% DRB formulation having 2.3 times more fiber than the control formulation. Fiber can improve texture and also provide stability to the product during processing, while incorporation of fibers into bakery products can also help control glycemia. The findings might agree with that of Alamsyah et al.^32^ reported that rice bran flour (RBF) is rich in dietary fibers and ash, thus making it fit for developing high-fiber cakes. Along with that, the fiber and ash content of the cake increased in proportion to the addition of RBF, thereby confirming its good fortifying potential toward enhancing the nutritional value of food products.

As shown in Table 2, the lowest energy value was recorded in the cake formulated with 40% rice bran (439.17 kcal/100 g), whereas the control cake exhibited the highest energy value (493.79 kcal/100 g). This reduction in energy content is primarily attributed to the partial replacement of corn oil with rice bran powder. Since fat provides 9 kcal/g, while carbohydrates and proteins contribute approximately 4 kcal/g and dietary fiber has limited metabolizable energy, the substitution resulted in a lower overall caloric density. Moreover, as indicated in Table 1, rice bran contains comparatively lower fat content and higher levels of carbohydrates and dietary fiber than wheat germ and pumpkin seeds. Therefore, reducing the lipid proportion in the formulation directly contributed to the decreased energy values observed in the rice bran cake. These findings are consistent with da Rocha Lemos Mendes et al.^31^ reported that the cake formulated with 40% defatted rice bran (DRB) had a reduced energy value (280.5 kcal/100 g) compared to the control cake and those containing 20% and 30% DRB (387.9, 363.3, and 341.5 kcal/100 g, respectively).

Table 2 illustrates the levels of bioactive compounds in the different cake formulations, including total phenolic content (TPC), total flavonoid content (TFC), and DPPH radical scavenging activity. The cake in which 60% of the fat was replaced with wheat germ (WG) exhibited the highest TPC (70.28 mg GAE/100 g) and TFC (11.99 mg RE/100 g). This observation is consistent with the raw material composition presented in Table 1, where wheat germ showed markedly higher phenolic and flavonoid contents compared to pumpkin seeds and rice bran. These results indicate that a substantial proportion of WG phenolic compounds was retained after baking, contributing to the enhanced bioactive profile of the final cake product. Conversely, the cake formulated with 40% rice bran (RB) as a fat replacer demonstrated the highest DPPH radical scavenging activity (58.65%), despite exhibiting a comparatively lower total phenolic content. This observation indicates that antioxidant activity is not solely dependent on total phenolic concentration but also on the qualitative composition and reactivity of individual bioactive compounds. As shown in Table 1, rice bran exhibited the strongest DPPH scavenging activity among the raw materials, which may be attributed to its richness in γ-oryzanol, ferulic acid, tocopherol, and tocotrienol compounds, well known for their potent free radical scavenging capacity. Moreover, the antioxidant effectiveness of rice bran in the cake matrix may result from synergy^35,36^. Similar findings were reported by Zolqadri et al.^37^ reported that sponge cakes fortified with 2% rice bran protein isolate exhibited significantly higher TPC and DPPH radical scavenging activity compared to control samples (*p* < 0.05), indicating a positive relationship between phenolic content and antioxidant capacity in baked products. This suggests that a considerable proportion of phenolic compounds can withstand thermal processing and remain functionally active after baking. Similarly, Manpreet Kaur and Sonika Sharma^38^ demonstrated that cakes supplemented with 20% raw pumpkin seeds flour exhibited higher antioxidant activity compared to roasted forms and refined wheat flour controls. This difference may be attributed to the degradation of thermolabile phenolic compounds during roasting, which can reduce the availability of active hydrogen-donating groups responsible for radical scavenging. In contrast, raw pumpkin seed flour retains a greater proportion of native bioactive compounds, thereby enhancing antioxidant performance in baked systems. These findings emphasize that both the intrinsic composition of the ingredient and the extent of prior thermal processing significantly influence antioxidant retention in bakery products.

Overall, although baking can reduce the absolute concentration of phenolic compounds due to thermal degradation and oxidation, wheat germ and rice bran retain considerable antioxidant activity. This may be explained by the presence of relatively heat-stable phenolic fractions and lipid-soluble antioxidants (e.g., tocopherols and γ-oryzanol), as well as potential protective interactions within the food matrix, such as binding to proteins or entrapment within dietary fiber networks. Consequently, the antioxidant performance of the cakes reflects not only the initial phenolic content but also the stability, composition, and matrix interactions of bioactive compounds present in the original raw materials^35^.

### Minerals and vitamins content of cake samples

Table 3 illustrates the mineral composition of the cake samples. The cake containing 60% wheat germ (WG) as a fat replacer showed higher levels of calcium, sodium, and copper (158.83, 193.27, and 11.93 mg/100 g, respectively) compared to the cakes with 60% pumpkin seeds (PS) and 40% rice bran (RB). In contrast, the cake containing 40% RB was superior in terms of magnesium, phosphorus, potassium, manganese, and selenium (466.43, 1210.88, 1194.62, 9.94, and 16.41 mg/100 g, respectively) compared to the other two formulations (60% PS and 60% WG). On the other hand, cakes prepared with 60% PS as a fat replacer recorded the highest iron and zinc content (14.60 and 9.82 mg/100 g) among all cake samples. These differences can be mechanistically explained by the intrinsic mineral composition of the substituted raw materials and the concentration effect resulting from partial fat replacement. Since corn oil contains negligible mineral content, its substitution with mineral-rich cereal by-products proportionally increased the ash and mineral density of the cake matrix. Wheat germ is naturally rich in calcium and copper due to its role as the embryo of the grain, where minerals are stored to support germination. Similarly, rice bran, being derived from the outer grain layers, is particularly rich in magnesium and phosphorus, largely associated with phytic acid complexes that remain relatively stable during baking. The elevated potassium and manganese levels in RB cakes may also reflect the mineral concentration in the bran fraction. In contrast, pumpkin seeds, as oilseeds, are well known for their high iron and zinc content, which explains their predominance in PS-enriched cakes. The structural association of these trace minerals with seed storage proteins may further contribute to their retention during thermal processing.

**Table 3:** Minerals and vitamins contents of cake samples

Component	cake samples			
	Control sample	60% WG	60% PS	40% RB
Minerals content (mg/100 g)				
Ca	69.14 ± 0.91 ^d^	158.83 ± 1.45^a^	84.67 ± 0.78 ^c^	92.86 ± 0.82^b^
Fe	5.08 ± 0.58 ^d^	10.75 ± 0.45^c^	14.60 ± 0.22^a^	12.64 ± 0.13^b^
Mg	153.91 ± 1.41 ^d^	287.48 ± 1.31^c^	354.61 ± 1.36^b^	466.43 ± 1.55^a^
P	539.65 ± 1.79 ^d^	774.04 ± 1.49^c^	1008.16 ± 1.61^b^	1210.88 ± 2.34^a^
K	598.17 ± 1.11 ^d^	994.26 ± 1.27^b^	841.63 ± 1.82^c^	1194.62 ± 1.89^a^
Zn	4.13 ± 0.16^c^	6.45 ± 0.14^b^	9.82 ± 0.11^a^	6.52 ± 0.20^b^
Mn	4.89 ± 0.11 ^d^	8.87 ± 0.07^b^	8.09 ± 0.08^c^	9.94 ± 0.21^a^
Na	39.17 ± 0.84^c^	193.27 ± 0.85^a^	114.54 ± 0.79^b^	118.43 ± 0.74^b^
Cu	10.23 ± 0.19^b^	11.93 ± 0.10^a^	11.55 ± 0.08^a^	11.35 ± 0.16^ab^
Se	10.08 ± 0.27^b^	10.54 ± 0.29^b^	10.42 ± 0.11^b^	16.41 ± 0.20^a^
Vitamin content (mg/100 g)				
Vitamin E	0.23 ± 0.02 ^d^	1.13 ± 0.07^a^	0.48 ± 0.03 ^c^	0.63 ± 0.04^b^
Vitamin B1	0.24 ± 0.01^b^	0.47 ± 0.04^a^	0.27 ± 0.04^b^	0.45 ± 0.02^a^
Vitamin B2	0.36 ± 0.03^b^	0.44 ± 0.03^a^	0.38 ± 0.02^ab^	0.38 ± 0.03^ab^
Vitamin B6	0.11 ± 0.02^b^	0.45 ± 0.04^a^	0.13 ± 0.03^b^	0.42 ± 0.02^a^

*60% WG* cake containing 60% wheat germ, *60% PS* cake containing 60% pumpkin seeds, *40% RB* cake containing 40% rice bran.

Data are presented as mean ± SD (*n* = 3). Different letters indicate significant differences at *p* < 0.05.

Although the absolute mineral values reported in this study are higher than those in some previous reports, this discrepancy can be attributed to the substantially higher substitution levels used in the present formulations. For instance, Ismeal et al.^39^ reported calcium, potassium, magnesium, and zinc contents of 98.89, 89.44, 469.55, and 3.75 mg/100 g, respectively, in bread with 15% wheat germ, compared to 46.27, 46.91, 176.22, and 0.98 mg/100 g in the control. Alves et al.^40^, reported rice bran contents in phosphorus (2933 mg/100 g), magnesium (1029 mg/100 g), potassium (211 mg/100 g), and calcium (56 mg/100 g), while Mahmoud et al.^41^ reported wheat germ as rich in calcium (1499.89 mg/100 g), magnesium (321.40 mg/100 g), and sodium (233.98 mg/100 g). Additionally, Olugbuyi et al.^42^ stated that Sample 1 contained 78.50% OFSP, 20% defatted sesame, and 1.60% rice bran, and that it showed the highest amounts of sodium (482.14 mg/g), calcium (48.14 mg/g), magnesium (122.7 mg/g), phosphorus (382.11 mg/g), and potassium (2682.11 mg/g), while Sample 2 was stated to have the highest amount of iron at 17.83 mg/100 g. Variations in raw material composition, geographic origin, processing, and analytical techniques are likely contributors to these differences. These findings support the mineral enrichment trends observed in the present study and confirm the effectiveness of high-level fat replacement with wheat germ, rice bran, and pumpkin seed powders in enhancing the micronutrient density of baked products.

Table 3 presents the vitamin composition of the different cake samples. Incorporation of 60% wheat germ (WG), 60% pumpkin seeds (PS), and 40% rice bran (RB) as fat replacers significantly enhanced the vitamin content compared to the control cake. Vitamin E content increased notably. The highest value was observed in the cake containing 60% WG (1.13 mg/100 g), followed by 40% RB (0.63 mg/100 g) and 60% PS (0.48 mg/100 g), while the control cake recorded 0.23 mg/100 g. A similar trend was observed for vitamin B1, reaching 0.47 mg/100 g in the 60% WG cake and 0.45 mg/100 g in the 40% RB cake, compared to 0.24 mg/100 g in the control. Vitamin B2 content also improved, with 0.44 mg/100 g in the 60% WG cake and 0.38 mg/100 g in cakes containing 60% PS or 40% RB. Vitamin B6 increased from 0.11 mg/100 g in the control cake to 0.45 mg/100 g in the 60% WG cake. The higher vitamin content observed in wheat germ enriched cakes may not only be attributed to the intrinsic vitamin composition of wheat germ but also to the potential protective role of its natural lipids and antioxidant compounds during baking. Wheat germ contains endogenous antioxidants, including tocopherols and phenolic compounds, which may reduce oxidative degradation under thermal processing conditions. Additionally, the residual lipid fraction may contribute to protecting fat-soluble vitamins, particularly vitamin E, from thermal and oxidative losses. This protective effect could partly explain the improved vitamin retention observed in the WG formulations compared to the control^43^.

Although these vitamin levels appear lower than some values reported in previous studies. Youssef^44^ observed 5.70 mg/100 g of vitamin E in biscuits fortified with 20% wheat germ, while Catzeddu et al.^43^ reported 54.5 µg/g (≈5.45 mg/100 g) in wheat germ-enriched pasta. This discrepancy can be explained by differences in product formulation, baking conditions, and wheat germ proportion. Cakes are baked for longer and at higher temperatures than pasta or biscuits. This can break down heat-sensitive vitamins like E and B-complex. Moreover, differences in moisture content and analytical methods may also contribute to variations in reported values. Despite these differences, the current results clearly demonstrate that the inclusion of functional ingredients, particularly wheat germ, significantly enhances the vitamin profile of cakes. This improvement increases their nutritional value and supports efforts to combat micronutrient deficiencies, highlighting the potential of these formulations as functional foods. These findings align with earlier reports emphasizing wheat germ as a rich source of vitamin E, thiamine (B1), and riboflavin (B2)^45,46^, confirming the effectiveness of incorporating nutrient-dense ingredients in baked products.

### Physical properties of cake samples

The physical properties, namely weight, volume, and specific volume, are essential quality indicators for cake samples, as they strongly influence consumer preference and overall acceptability. These characteristics are closely linked to the type and amount of fat incorporated into the batter, since fat enhances aeration and contributes to the stabilization of air bubbles formed during mixing, which in turn affects cake texture and volume^47^.

**Fig. 2:**
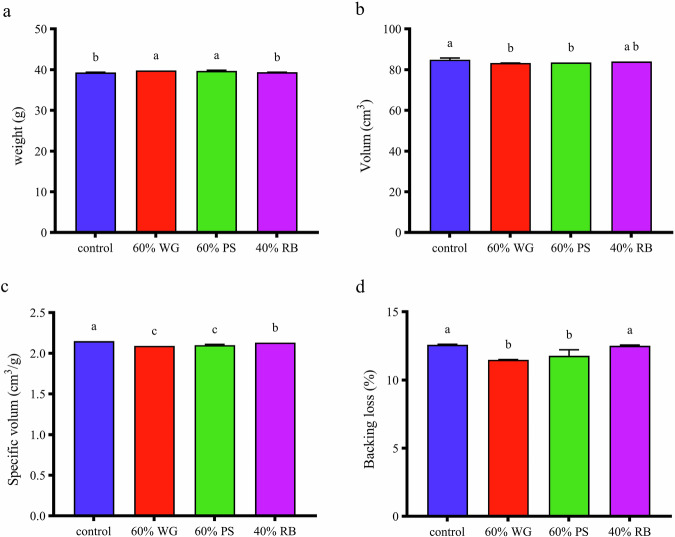
Physical properties of cake samples. (**a**) weight (g), (**b**) volume (cm^3^), (**c**) specific volume (cm^3^/g), and (**d**) baking loss (%). 60% WG cake containing 60% wheat germ, 60% PS cake containing 60% pumpkin seeds, 40% RB cake containing 40% rice bran. Data are presented as mean ± standard deviation (*n* = 3). Error bars represent standard deviation. In some cases, error bars are not visually distinguishable due to very small variation.

The physical properties of the cake samples, including baking loss, weight, volume, and specific volume, are presented in Fig. 2a–d. The control cake exhibited the highest baking loss, whereas cake samples with 60% wheat germ (WG), 60% pumpkin seed powder (PS), and 40% rice bran (RB) showed a significant decrease in baking loss (*p* < 0.05). This reduction can be explained by the high water- and oil-holding capacities of the raw materials (Table 1), particularly wheat germ, which exhibited the highest water-holding capacity (264.00%) and oil-holding capacity (236.67%) compared to pumpkin seeds and rice bran. These properties help retain moisture and fat during baking, reduce baking losses, and enhance texture and mouthfeel^37^.

Cakes containing 60% WG and PS as a fat replacer showed a significant increase in weight compared to the control and 40% RB cakes (*p* < 0.05), likely due to higher water retention associated with elevated protein (32% in PS, 23.45% in WG) and fiber contents (Table 1). In contrast, 40% RB cakes had lower weight, which can be explained by the lower protein content (12.14%) and slightly lower water-binding capacity despite higher fiber levels, consistent with Alamsyah et al.^32^.

Volume and specific volume were significantly reduced in cakes with WG, PS, and RB compared to the control (*p* < 0.05). This decrease is likely caused by (i) the (i) dilution of gluten-forming proteins, which weakens the gas-holding network, and (ii) the increased fiber content that absorbs water and limits air bubble expansion, as also noted by Ike et al.^48^. The high water- and oil-holding capacity of wheat germ helps retain moisture but contributes to a denser crumb, while pumpkin seeds, with lower WHC/OHC and higher protein and fat, create a firmer structure with reduced volume.

Overall, the observed changes in the physical properties of the cakes can be directly linked to the compositional differences of the raw materials (Table 1). High protein and fiber levels in PS and WG promote moisture retention and weight gain but interfere with gluten network development, reducing volume and increasing firmness. Rice bran, although high in fiber, has comparatively lower protein content, partially explaining its intermediate effects on cake structure and baking loss. These findings align with previous studies demonstrating that fibrous or protein-rich flour additions can increase weight, reduce volume, and alter texture in baked products (Zolqadri et al.^37^; Alamsyah et al.^32^; Ike et al^48^.).

### Color measurements of cake samples

Table 4 presents the crust and crumb color parameters (L*, a*, b*) of the various cake samples. The control cake exhibited the highest crust lightness (L* = 72.38), indicating a relatively pale surface. In contrast, the cake prepared with 60% wheat germ (WG) as a fat replacer showed the lowest crust L* value (56.60), reflecting a noticeably darker crust. This reduction in lightness can be mechanistically attributed to intensified Maillard reactions and caramelization during baking, promoted by the higher protein and lower sugar content of wheat germ. The increased availability of amino groups and sugars enhances non-enzymatic browning, leading to darker crust formation.

**Table 4:** Color measurements of cake samples

Color values	Crust color Crumb color					
	L^*^	a^*^	b^*^	L^*^	a^*^	b^*^
Samples						
Control cake	72.38 ± 0.20^a^	8.01 ± 0.29^c^	10.81 ± 0.42 ^d^	56.87 ± 1.92^c^	13.98 ± 0.51^a^	21.05 ± 0.91^c^
60% WG	56.60 ± 0.40 ^d^	12.49 ± 0.27^b^	20.89 ± 0.30^c^	64.92 ± 1.71^b^	12.31 ± 0.50^b^	25.39 ± 0.98^b^
60% PS	58.98 ± 0.09^c^	14.38 ± 0.30^a^	23.86 ± 0.49^b^	61.19 ± 1.16^b^	12.82 ± 0.31^b^	22.46 ± 0.73^c^
40% RB	62.53 ± 0.22^b^	7.26 ± 0.43^c^	26.97 ± 0.35^a^	68.44 ± 0.88^a^	8.43 ± 0.66^c^	30.08 ± 1.36^a^

Data are presented as mean ± SD (*n* = 3). Different letters indicate significant differences at *p* < 0.05.

*60% WG* cake containing 60% wheat germ, *60% PS* cake containing 60% pumpkin seeds, *40% RB* cake containing 40% rice bran.

For the crumb, the cake containing 40% rice bran (RB) displayed the highest L* value, suggesting a lighter internal appearance, while other samples exhibited slightly darker crumbs. This phenomenon may be explained by the dilution effect of lipid removal combined with the relatively lighter internal matrix formed by rice bran particles dispersed within the batter system. This phenomenon may be explained by the dilution effect of lipid removal combined with the relatively lighter internal matrix formed by rice bran particles dispersed within the batter system. The increased redness in PS cakes may be associated with natural pigments and phenolic compounds present in pumpkin seeds, which can undergo thermal transformations and contribute to brown-red chromatic development. Crumb redness followed a similar trend, with the control cake showing the highest a* value and the 40% RB cake the lowest, suggesting that fiber-rich formulations may limit pigment concentration and browning intensity within the internal structure.

For yellowness (b*), the cake containing 40% RB showed the highest b* values for both crust (26.97) and crumb (30.08), indicating a more pronounced yellow hue, while the control cake recorded the lowest values. The enhanced yellowness in RB cakes may be linked to naturally occurring pigments and phenolic derivatives in the bran layer, as well as to structural light scattering effects caused by increased dietary fiber content. Fiber particles can influence light reflectance properties of the crumb, thereby modifying perceived color parameters^35^. According to da Rocha Lemos Mendes et al.^31^ the addition of defatted rice bran (DRB) can significantly influence crumb color, often resulting in darker (lower L*) and redder (higher a*) crumbs. The crumb color is primarily affected by the type and composition of raw materials used, whereas crust color is largely determined by chemical reactions such as Maillard browning and caramelization during baking. Similar effects on crumb color due to rice bran incorporation have been reported in other bakery products. Thus, the impact of rice bran on cake color can vary depending on its concentration, type, and interaction with other ingredients. On the other hand, Ismeal et al.^39^ reported that the addition of wheat germ (WG) led to a decrease in *L* values (lightness) and an increase in a (redness) and b* (yellowness) values for both crumb and crust color. These changes were primarily attributed to the inherent natural color of wheat germ. Additionally, the high amino acid and sugar content in WG likely enhanced Maillard reactions during baking, contributing to increased browning and red tones. At inclusion levels up to 10%, the loaf’s color remained similar to that of the control bread, likely due to the presence of carotenoids in WG. The b* value (brownness) also rose with WG supplementation, as the elevated protein and sugar content intensified the Maillard reaction. Furthermore, it was noted that the bread’s surface became progressively darker with higher levels of WG addition, particularly up to 30%. According to Mahmoud et al.^41^, increasing the level of wheat germ (WG) in biscuits led to a significant (*P* ≤ 0.05) increase in their darkness, as evidenced by lower L* values. This indicates that all biscuits containing WG appeared darker than the control. The redness (a*) values did not show significant differences (*P* ≤ 0.05) among the various samples. A slight reduction in yellowness (b*) values was observed as WG levels increased. Likewise, the hue angle slightly decreased with higher WG content, pointing to a browner color in the biscuits. Chroma, which reflects color intensity, also declined with increased WG addition compared to the control. This change in color is likely due to a decrease in brightness and an increase in redness. The darkening of the enriched biscuits could be attributed to Maillard reactions, caramelization, higher protein content, or pH changes.

Overall, crust color was predominantly governed by non-enzymatic browning reactions, whereas crumb color was more influenced by ingredient composition and structural characteristics. These findings confirm that replacing fat with WG, PS, or RB alters color development through combined effects of intrinsic pigments and thermal reaction kinetics.

### Texture profile analysis of cake samples

**Fig. 3:**
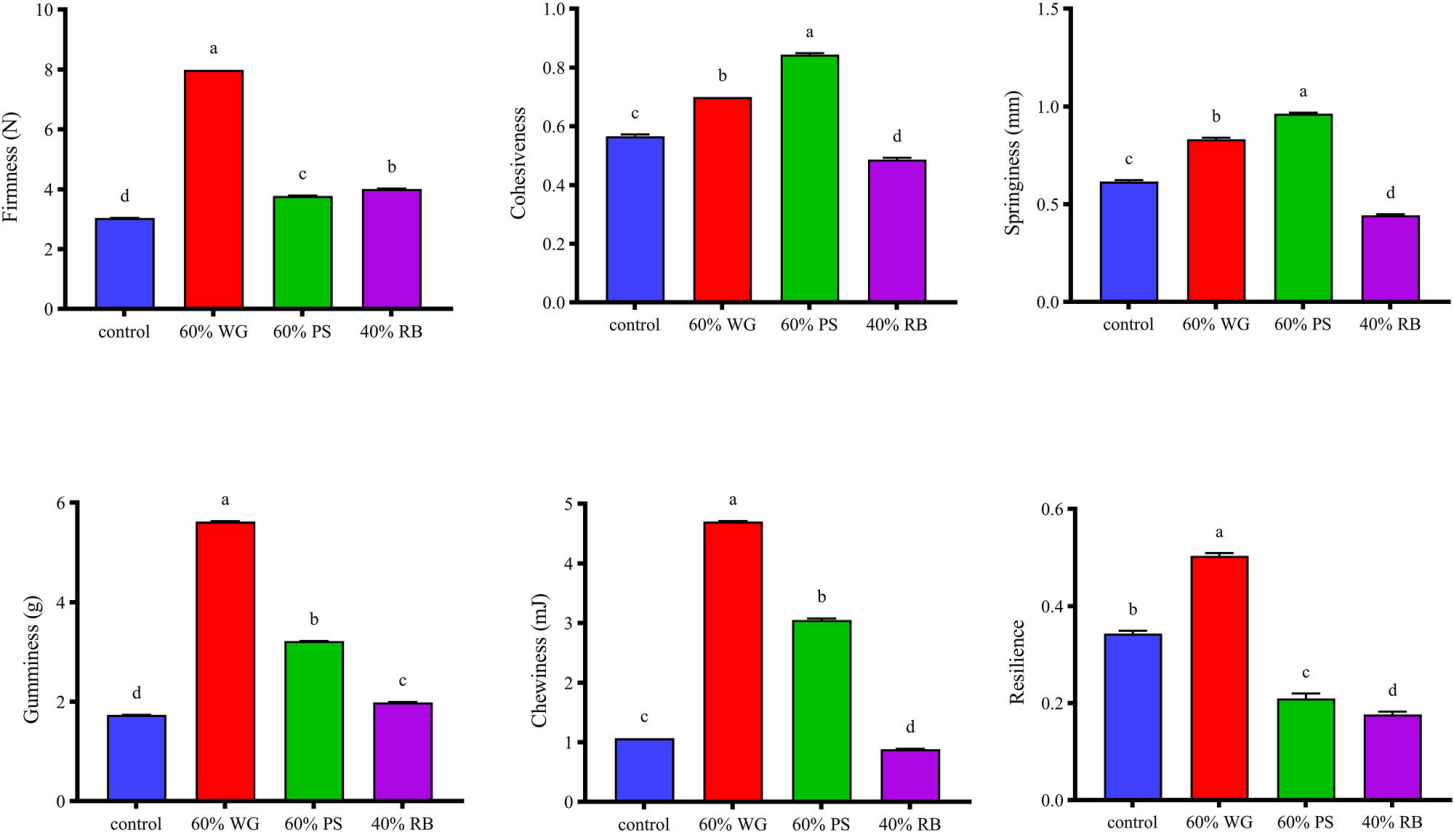
Texture profile analysis of cake samples. 60% WG cake containing 60% wheat germ, 60% PS cake containing 60% pumpkin seeds, 40% RB cake containing 40% rice bran. Data are presented as mean ± standard deviation (*n* = 3). Data are presented as mean ± standard deviation (*n* = 3). Error bars represent standard deviation. In some cases, error bars are not visually distinguishable due to very small variation.

The Texture Profile Analysis (TPA) of the cake samples is presented in Fig. 3. The cake prepared with 60% wheat germ (WG) exhibited the highest values for firmness, gumminess, chewiness, and resilience, which can be attributed to the high protein and fiber content, as well as the superior water- and oil-holding capacities of wheat germ (Table 1). These components promote a denser crumb structure, retain moisture, and strengthen the matrix, leading to a firmer texture.

In contrast, the cake containing 60% pumpkin seeds (PS) as a fat replacer showed significantly higher cohesiveness and springiness compared to other samples. This is likely due to the balanced protein-lipid composition of pumpkin seeds, which enhances crumb elasticity and structural cohesion. Meanwhile, the cake with 40% rice bran (RB) recorded the lowest values of cohesiveness, chewiness, and resilience, indicating a softer and less elastic texture. The lower hydration capacity and reduced protein content of rice bran result in a less dense matrix, limiting gas retention during baking and yielding a softer crumb.

These findings are consistent with previous reports that high-fiber and high-protein ingredients increase firmness and chewiness while potentially reducing springiness, whereas lower concentrations produce softer, more elastic textures (Zolqadri et al.^37^; Mahmoud et al.^41^). Overall, the texture profile of the cakes is strongly influenced by the composition and functional properties of the fat replacers, highlighting their role in shaping cake structure and mouthfeel.

### Sensory evaluation of cake samples

**Fig. 4:**
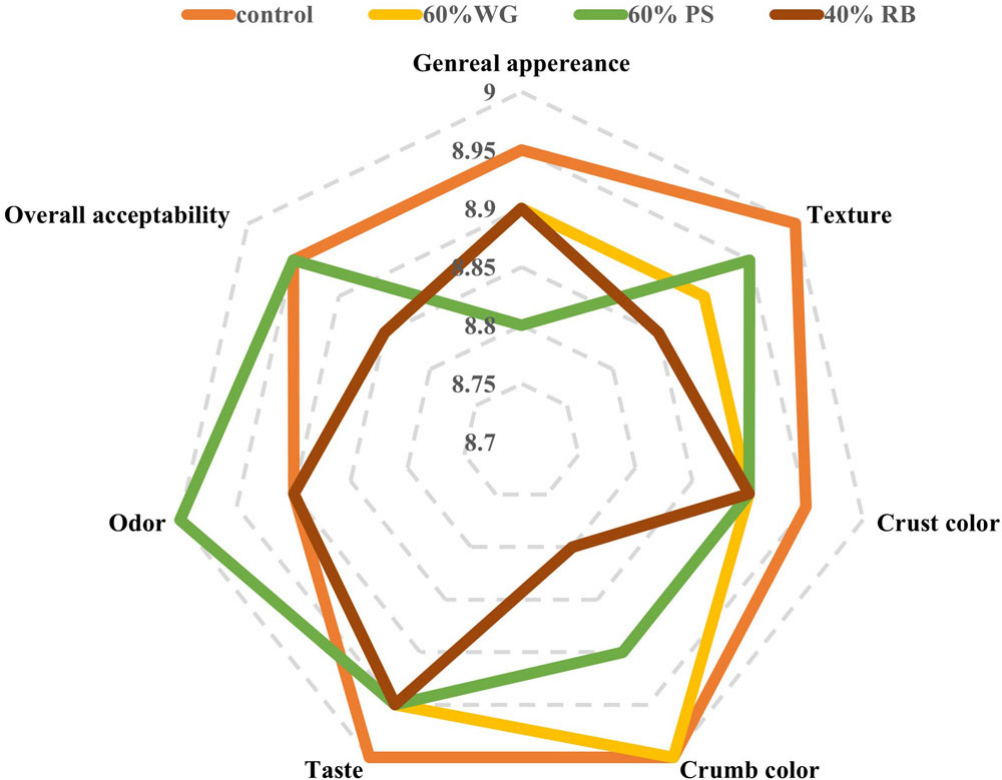
Sensory evaluation of cake samples. 60% WG cake containing 60% wheat germ, 60% PS cake containing 60% pumpkin seeds, 40% RB cake containing 40% rice bran.

Results of sensory evaluation (Table 5 and Fig. 4) revealed no significant differences (*P* > 0.05) among the control cake and those formulated with 60% wheat germ (WG), 60% pumpkin seeds (PS), and 40% rice bran (RB) in terms of general appearance, texture, crust color, crumb color, taste, odor, and overall acceptability. Duncan’s Multiple Range Test confirmed that all four samples were statistically comparable across all evaluated attributes, as indicated by identical superscript letters within each row (Table 5). Although the control sample scored slightly higher in some attributes, all fortified cakes received comparably high ratings, indicating that high-level fat replacement did not adversely affect sensory quality. The maintained acceptability may be attributed to the functional properties of the substituted ingredients, particularly their ability to retain moisture and contribute desirable flavor characteristics, such as the nutty notes associated with wheat germ and pumpkin seeds. These findings suggest that incorporating WG, PS, and RB can enhance the nutritional profile of cakes without compromising consumer perception.

**Table 5:** Sensory evaluation of cake samples

Sensory evaluation	cake samples			
	Control sample	60% WG	60% PS	40% RB
General appearance	8.95 ± 0.16^a^	8.90 ± 0.21^a^	8.80 ± 0.35^a^	8.90 ± 0.21^a^
Texture	9.00 ± 0.00^a^	8.90 ± 0.21^a^	8.95 ± 0.16^a^	8.85 ± 0.34^a^
Crust color	8.95 ± 0.16^a^	8.90 ± 0.21^a^	8.90 ± 0.21^a^	8.90 ± 0.21^a^
Crumb color	9.00 ± 0.00^a^	9.00 ± 0.00^a^	8.90 ± 0.21^a^	8.80 ± 0.35^a^
Taste	9.00 ± 0.00^a^	8.95 ± 0.16^a^	8.95 ± 0.16^a^	8.95 ± 0.16^a^
Odor	8.90 ± 0.32^a^	8.90 ± 0.32^a^	9.00 ± 0.00^a^	8.90 ± 0.32^a^
Overall acceptability	8.95 ± 0.16^a^	8.85 ± 0.24^a^	8.95 ± 0.16^a^	8.85 ± 0.34^a^

*60% WG* cake containing 60% wheat germ, *60% PS* cake containing 60% pumpkin seeds, *40% RB* cake contain-ing 40% rice bran.

Data are presented as mean ± SD (*n* = 30). Different letters indicate significant differences at *p* < 0.05.

Similar observations have been reported in bakery products enriched with rice bran and wheat germ, where moderate substitution levels did not significantly affect sensory scores (Alamsyah et al.^32^; Mahmoud et al.^41^) In addition, pumpkin seed incorporation has been associated with improved flavor and overall acceptability in cakes (Manpreet Kaur and Sonika Sharma^38^; Ike et al.^48^). These reports support the present results and confirm the feasibility of using these by-products as functional fat replacers in bakery formulations.

### Microbial quality of cake samples

**Fig. 5:**
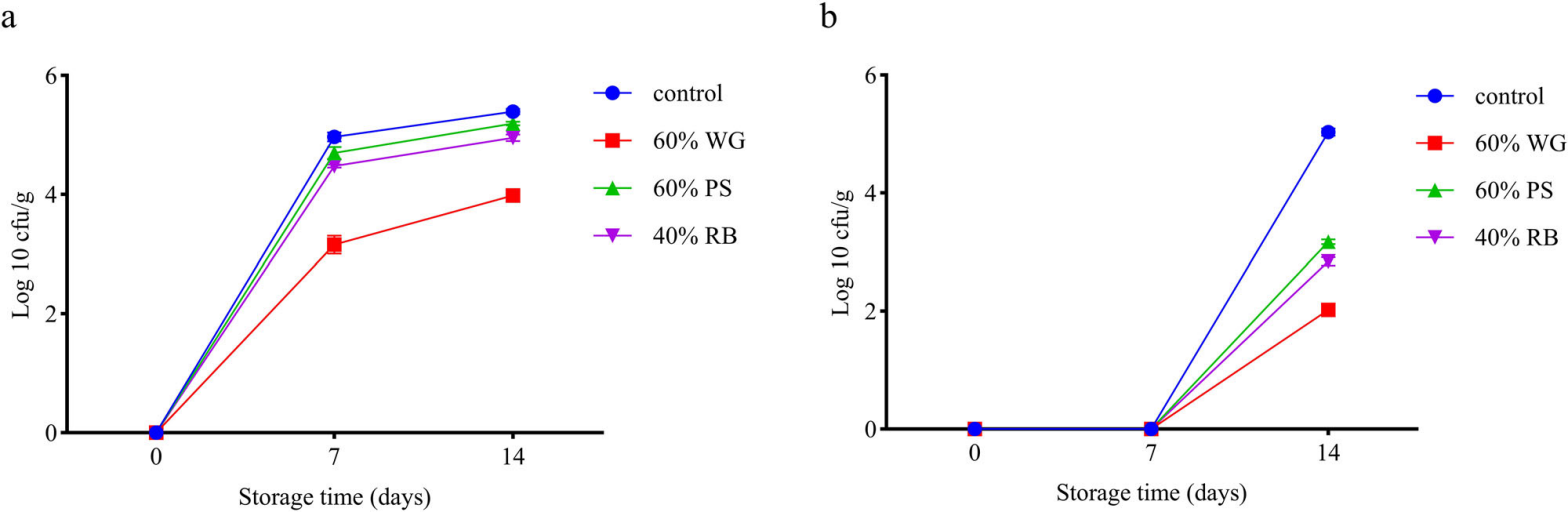
Microbial quality of cake samples. **a** Total bacteria count and **b** Mold & yeast count, *60% WG* cake containing 60% wheat germ, *60% PS* cake containing 60% pumpkin seeds, *40% RB* cake containing 40% rice bran. Data points represent mean ± standard deviation (*n* = 3).

Baked goods are susceptible to spoilage from post-baking contamination, particularly when stored improperly for extended periods. Mold growth is among the most frequent causes of spoilage in these products. A food item is typically regarded as spoiled when mold or bacterial counts exceed 10⁷ CFU/g or 10⁵ CFU/g, respectively^49^.

(Figure 5a, b) illustrates the total bacterial and mold & yeast counts (Log 10 cfu/g) in the cake samples stored at room temperature for two weeks, used as an indicator of the product’s shelf life. Initially (at zero time), none of the samples exhibited detectable microbial growth, reflecting proper hygiene and effective baking. However, microbial levels increased progressively after one and two weeks of storage. The control cake recorded the highest bacterial count (5.39 Log 10 cfu/g) and mold and yeast count (5.03 Log 10 cfu/g) by the end of the storage period, indicating a higher vulnerability to spoilage. Conversely, cakes formulated with 60% wheat germ (WG), 60% pumpkin seeds (PS), and 40% rice bran (RB) as a fat replacer showed significantly reduced microbial growth. Among these, the cake with 60% WG exhibited the strongest resistance to microbial spoilage, having the lowest total bacterial (3.98 Log 10 cfu/g) and mold & yeast (2.02 Log 10 cfu/g) counts at the end of storage. This enhanced microbial resistance may be attributed to the higher content of bioactive compounds like total phenolics and flavonoids in wheat germ, as shown in Table 1, compared to PS and RB. These findings suggest that incorporating WG, PS, and RB can improve the microbial stability of baked products, likely due to their antimicrobial properties or their effect on water activity. In addition to the antimicrobial effects of bioactive compounds, the observed reduction in microbial counts may also be attributed to the decreased water activity (aw) of the cake matrix. The high water-binding capacity of dietary fibers present in wheat germ, pumpkin seeds, and rice bran reduces the availability of free water, thereby creating a less favorable environment for microbial growth during storage^48^. These findings are in partial agreement with those reported by Ike et al.^48^ who found that the bacterial and fungal counts (CFU/g) in baked cakes ranged from 1.40 × 10² to 2.99 × 10³ and from 2.50 × 10¹ to 2.40 × 10², respectively. The control cake showed the highest microbial counts, whereas CF6 (the cake formulated with 60% pumpkin seed flour) exhibited the lowest bacterial and fungal counts across both storage durations under ambient and refrigerated conditions. Refrigeration had a suppressive effect on microbial cell replication, resulting in reduced growth rates and lower microbial loads in samples stored at cooler temperatures compared to those kept at room temperature. The study also revealed a correlation between moisture content, blending ratios, and microbial counts; lower moisture content and higher PSF levels resulted in reduced microbial growth. This is likely due to PSF’s low moisture and high oil content, which inhibits aerobic microorganisms by restricting oxygen flow, creating an unfavorable environment for their growth. In the study by da Rocha Lemos Mendes et al.^31^, cakes formulated with 20%, 30%, and 40% defatted rice bran demonstrated acceptable microbiological quality. Levels of B. cereus, thermotolerant coliforms, molds, and yeasts were all below 100 CFU/g, and Salmonella was not detected in 25 g samples. The low counts of thermotolerant coliforms suggest that good manufacturing practices were followed during production. Furthermore, none of the samples showed B. cereus contamination exceeding 100 CFU/g. The present findings are also supported by recent studies demonstrating that plant-derived functional ingredients rich in bioactive compounds can improve the microbial stability and extend the shelf life of bakery products while enhancing their nutritional quality. These natural ingredients have attracted increasing attention as sustainable alternatives to synthetic preservatives in the development of clean-label bakery products^50,51^.

One limitation of this study is that the mineral content of the raw materials was not directly analyzed prior to formulation. Therefore, the mineral composition reported reflects the combined effect of ingredient incorporation and baking. Future studies should include direct analysis of the raw materials to more precisely quantify their individual contributions and to evaluate the bioavailability of these minerals within the cake matrix. This would provide a more accurate assessment of the nutritional and functional value of the fat replacers. Another limitation is that the effectiveness of the stabilization process for wheat germ (WG) and rice bran (RB) was not directly evaluated through oxidative indicators such as acid value or peroxide value. Thus, the effectiveness of the stabilization process was inferred based on established literature and prior experience with similar materials. Future studies should include direct measurement of enzyme activity and lipid oxidation before and after stabilization to quantitatively confirm its efficiency. This would further strengthen the scientific basis for the use of stabilized WG and RB as natural fat replacers and support formulation optimization.

This study demonstrates the potential application of wheat germ, pumpkin seed powder, and rice bran by-products as multi-functional fat replacers in cake formulations. Their incorporation was associated with improved nutritional attributes, including increased protein, dietary fiber, vitamins, minerals, antioxidant capacity, and enhanced shelf stability, without adversely affecting sensory acceptability. Specifically, the inclusion of 60% wheat germ was associated with higher vitamin and antioxidant levels, while 40% rice bran contributed to increased mineral content and antioxidant activity. In addition, 60% pumpkin seeds powder improved protein and fiber levels. These findings suggest that such by-products may contribute to improving the nutritional quality of bakery products while supporting the sustainable utilization of food processing residues. Therefore, the incorporation of these by-products as fat replacers can be considered a promising strategy for the development of healthier, nutrient-enriched bakery products. However, further studies are recommended to evaluate large-scale processing conditions and long-term storage stability. Overall, the results provide supportive evidence for the feasibility of using these by-products as functional fat replacers in cake samples.

## Methods

### Materials

Bran from the Sakha 106 rice variety was obtained from the Rice Research and Training Center (RRTC) at Sakha, Kafr El-Sheikh, Egypt. Other ingredients, including wheat germ, pumpkin seeds (*Cucurbita pepo L*.), 72% extraction wheat flour, sugar, baking powder, corn oil, fresh eggs, skimmed milk powder, and vanillin powder, were purchased from a local market in Giza, Egypt. All chemicals used in the present study were of analytical reagent grade.

### Wheat germ, pumpkin seeds, and rice bran flour Preparation

The collected wheat germ, pumpkin seeds, and rice bran were clean of any foreign materials completely.

### Stabilization of wheat germ and rice Bran

The stabilization of wheat germ (WG) and rice bran (RB) was carried out according to Malekian et al.^52^. This step is critical to inactivate lipase and other oxidative enzymes, which are naturally present in WG and RB and can rapidly degrade lipids, leading to rancidity and loss of nutritional quality. Specifically, 100 g of WG or RB was packed in a polyethylene microwave-safe bag and subjected to microwave heating in a preheated oven at 120 °C for 3 min. The procedure was repeated three times to ensure complete enzyme inactivation. Following stabilization, WG, RB, and pumpkin seeds were milled using a Moulinex grinder, passed through a 40-mesh sieve (420 µm), packed in polyethylene bags, and stored at −30 °C until further analysis. This protocol preserves the nutritional and functional properties of the ingredients, ensuring their suitability as natural fat replacers in cake samples.

### Cake samples preparation

Cake samples were prepared according to the specifications in Table 6, based on the method described by Bennion et al.^53^ with slight modifications. The control cake was formulated using corn oil as the sole fat source. In the experimental treatments, corn oil was partially replaced with natural fat substitutes, namely wheat germ (WG, 60%), pumpkin seeds powder (PS, 60%), and rice bran (RB, 40%). Different replacement levels were selected based on preliminary trials to achieve acceptable batter handling properties and sensory quality, ensuring comparable processing behavior among samples. Here, the replacement percentages indicate the proportion of the original corn oil weight that was substituted with the equivalent weight of the respective powder. For instance, 60% WG means that 60% of the corn oil weight in the control recipe (65 g) was replaced with 39 g of wheat germ powder. This clarification has also been added as a footnote in Table 6.

**Table 6:** Formulation of cake samples

Components	Cake samples			
	Control sample	60% WG	60% PS	40% RB
Wheat flour 72% ext.	100	100	100	100
Corn oil	65.00	26.00	26.00	39.00
WG	—	39.00	—	—
PS	—	—	39.00	—
RB	—	—	—	26.00
Sugar	85.00	85.00	85.00	85.00
Whole egg	85.00	85.00	85.00	85.00
Skimmed milk powder	3.00	3.00	3.00	3.00
Vanillin	0.60	0.60	0.60	0.60
Baking powder	3.80	3.80	3.80	3.80
Water (mL)	50.00	70.00	65.00	60.00

The fat replacement levels in the cake formulations are based on the weight of corn oil in the control recipe. For example, 60% WG (wheat germ) or 60% PS (pumpkin seeds powder) indicates that 60% of the corn oil (65 g) was replaced with 39 g of WG (wheat germ) or PS (pumpkin seeds powder), respectively, while 40% RB (rice bran) indicates that 40% of the corn oil was replaced with 26 g of RB.

Sugar and oil (or oil–fat replacer blends) were creamed in a mixer for 3 min at speed 5, after which eggs were incorporated for an additional 2 min of mixing. Flour, baking powder, and skim milk powder were then added and mixed for 4 min at speed 2. Water was added according to the water absorption capacity of each formulation to obtain a uniform batter consistency. The exact amounts of water used were 50 mL for the control, 70 mL for the 60% WG formulation, 65 mL for the 60% PS formulation, and 60 mL for the 40% RB formulation, as shown in Table 6. All samples were mixed under identical conditions (time and speed) to ensure comparable processing behavior. The prepared batter was poured into baking pans and baked in a preheated oven at 180 °C for 30 min. Cakes were allowed to cool, wrapped in polyethylene bags, and stored at room temperature (25 ± 2 °C) for up to 15 days. Samples were analyzed within 1 h of baking, and the remaining samples were analyzed at 7-day intervals during storage.

### Chemical composition

The raw materials and cake samples were analyzed for the chemical composition in accordance with the AOAC^54^ standards for ash, crude fiber, protein, fat, and moisture. The total carbohydrate content was calculated by difference. The energy content of the cake samples was determined using the AOAC^54^ formula:

$${Energy}\,{content}\,\left(K.{cal}\right)=4\left[{\rm{protein}}\left({\rm{g}}\right)+{carbohydrates}\,\left(g\right)\right]+[9* {fat}\left(g\right)]$$

### Extract preparation

Sample extraction was performed as described by Öztürk et al.^55^. In brief, 5 grams of sample were homogenized in 25 mL of 75% methanol, using a digital homogenizer (PRO25D, Thomas Scientific, USA). The homogenate was then centrifuged, using a Sigma 113 centrifuge (VWR International, Darmstadt, Germany) at 7000 rpm for 15 min at 4 °C. The supernatant was filtered through Whatman No. 1 filter paper and stored at 4 °C for further analysis of phenolic extracts.

### Total phenolic content determination

The total phenolic content determination was carried out according to the method of Abirami et al.^56^. A few words: 0.2 mL methanolic extract was mixed with 1:10 diluted Folin-Ciocalteu reagent and mixed for 4 min; then 0.8 mL 7.5% sodium chloride (w/v) was added to the mixture. The final incubation was at room temperature for 30 min and subsequently subjected to centrifugation at 5000 rpm for 10 min. The sample absorbance was read at 765 nm.

### Total flavonoid determination

Total phenolic compounds were estimated as described by Barros et al.^57^. Briefly, 0.5 mL of the extract was diluted with 2 mL of distilled water, followed by the addition of 150 and 150 μL of 5% sodium nitrite (NaNO₂). Six minutes later, 150 μL of 10% aluminum chloride (AlCl₃) was added, and after another 6 minutes, 2 mL of 4% sodium hydroxide (NaOH) solution was added to end the reaction, and the final volume was made up to 5 mL with distilled water. After that, the solution was allowed to stand for 15 min, and the absorbance was read at 510 nm using a PG T80+ spectrophotometer (England).

### Assessment of antioxidant activity

The evaluated antioxidant activity of extracts on their DPPH free radical scavenging model as per Brand-Williams et al.^58^. Various concentrations of extracts dissolved in methanol were added to glass test tubes containing 2 mL DPPH solution. The control was 2 mL of DPPH solution with 2 mL of methanol, while the blank was methanol alone for 1 mL. All mixtures were carried out in darkness for 30 min, and their absorbance was read at 517 nm using the PG T80+ spectrophotometer. Antioxidant activities were expressed as a percentage of radical scavenging activity.

$${Scavenging}\,{activity}\,\left( \% \right)=\left(\text{Abs control}-\text{Abs sample}/\text{Abs control}\right)\times 100$$

### Water holding capacity (WHC) and oil holding capacity (OHC)

Measurement of WHC and OHC was done according to the procedure described by Meriles et al.^59^. 10% w/v suspensions of samples were prepared in distilled water or corn oil, respectively, stirred, and left at room temperature for 30 min and then centrifuged at 2000 × *g* for 25 min. WHC and OHC values were expressed as the weight difference per gram of dry sample.

$${\rm{WHC\; \& \; OHC}}=\frac{\left({\rm{m}}2-{\rm{m}}1\right)}{{\rm{m}}1}$$

where m1 is weight of the dry sample and m2 is weight of the sediment.

### Analyses of mineral contents

The minerals P, Mg, Fe, K, Ca, Na, Cu, and Zn present in the analyzed raw materials and cake samples were determined by the standard procedure of AOAC^54^ using the method of ICP-OES Agilent 5100 VDV systems.

### Vitamin analysis

A reversed‑phase HPLC method adapted from Moreno and Salvado^60^ was used for vitamin analysis. Water-soluble vitamins were separated on a Nova-Pack C18 analytical column (150 × 3.9 mm, 4 µm) using an isocratic mobile phase of methanol and 0.05 M ammonium acetate buffer (ratio optimized according to reference conditions), with a flow rate of 2 mL/min and UV detection at 270 nm. Fat-soluble vitamins were separated on the same column using an isocratic mobile phase of methanol–acetonitrile (95:5, v/v), at the same flow rate of 2 mL/min, with UV detection at 285 nm. These chromatographic parameters ensure reproducible separation of the target vitamins.

### Physical properties of cake samples

Average weights (g) of each cake sample were calculated separately within an hour after baking. The volume of cake (cm³) was determined using the rapeseed displacement method, while the specific volume (cm³/g) was calculated as per the procedure given by AACC^61^ using the following equation:

$${Specific}\,{volume}\,({{cm}}^{3}/g)\,=\frac{{Volume}\left({{cm}}^{3}\right)}{{Weight}\,(g)}$$

### Color measurements of cake samples

Color assessment of cake samples was carried out as directed by McGuire^62^. The crust and crumb color readings were made by using an in-house tristimulus reflectance colorimeter (Model CS-400, Konica Minolta, Japan). The machine provided the following readings: L* (lightness—and *L* = 100 means white, whereas *L* = 0 means black), a* (chromaticity from green (−) to red (+)), and b* (chromaticity from blue (−) to yellow). Each sample was color-measured thrice, and means were calculated from these readings.

### Texture profile analysis

Texture parameters, including firmness, gumminess, chewiness, cohesiveness, springiness, and resiliency, were assessed with the TA-XT 2 Texture Meter (Texture Pro CT3 V1.2, Brookfield, Middleboro, USA), as described by Yuan and Chang^63^. The TPA measurements were done using a cylindrical probe of 25 mm diameter with a speed of 2 mm/s. Each sample was subjected to three measurements, and the average was taken.

### Sensory evaluation

After baking, cake samples were allowed to cool for 4 h at 25 ± 2 °C before sensory evaluation. All sensory evaluation procedures were conducted in accordance with the ethical standards of the institutional research committee. The protocol was approved by the Sensory Evaluation Ethics Committee (SEC-18), Food Technology Research Institute, Agricultural Research Center (ARC), Egypt. Written informed consent was obtained from all panelists prior to participation. The evaluation was conducted by thirty trained panelists (15 males and 15 females) from the Food Technology Research Institute, who were experienced in the sensory assessment of bakery products. Cake samples were served on odorless white disposable plates and presented in a randomized order. Panelists evaluated taste, aroma, texture, appearance, and overall acceptability using a 9-point hedonic scale (1 = dislike extremely, 5 = neither like nor dislike, and 9 = like extremely), following the method described by Bennion et al.^53^.

### Microbiological analysis

The microbiological quality of cakes stored for two weeks under ambient conditions was assessed via total fungal count from 1 g of sample using malt yeast agar, a medium proven to be an effective index for shelf life^64^. The aerobic plate count was also done using total count media, as per Sawanson et al.^65^.

### Statistical analysis

Statistical analysis was performed using SAS software (SAS/ETS, Version 9.1; SAS Institute Inc., Cary, NC, USA) SAS^66^. One-way analysis of variance (ANOVA) was applied to determine the effect of fat replacement on the studied cake parameters. Mean comparisons were conducted using Duncan’s Multiple Range Test, and differences were considered statistically significant at *p* < 0.05.

## Data Availability

The datasets generated in the current study are available on Figshare at 10.6084/m9.figshare.32226333.
